# Impact of varicocoelectomy on male semen parameters: A long‐term analysis of sperm quality and outcomes

**DOI:** 10.1111/andr.70075

**Published:** 2025-05-28

**Authors:** Max D. Sandler, Julio Yanes, Rohan Dureja, Vishal Ila, Aaron A. Gurayah, Adam D. Williams, David Miller

**Affiliations:** ^1^ Desai Sethi Urology Institute University of Miami Miller School of Medicine Miami Florida USA; ^2^ Department of Urology Weill Cornell Medical Center New York New York USA

**Keywords:** infertility, semen parameters, sperm motility, varicocoeles

## Abstract

**Background:**

Varicocoeles are common in 40% of men presenting with infertility. Semen parameters including sperm motility, low sperm count and sperm morphology are altered by the presence of varicocoeles. Though varicocoelectomy has been associated with improvements in these parameters, studies on long‐term outcomes are limited.

**Objectives:**

Analyze pre‐operative samples with those collected at multiple post‐operative timepoints to assess if sperm parameters continue to improve in the period following varicocoelectomy.

**Materials and methods:**

Adult men who underwent varicocoelectomy under a single surgeon from 2017 to 2023 with at ≥1 post‐operative semen analysis (SA) were included. Motile sperm count, progressive motility, round cells, semen volume, sperm concentration, sperm morphology, total motility and viability was collected from each patient's SA. Wilcoxon signed‐rank tests were used to compare medians of sperm parameters from pre‐operative baseline to 6, 12, 18 and 24 months follow up.

**Results:**

At 6 months, number of motile sperm increased from 4 to 8 million/mL (*p* < 0.0001), progressive motility increased from 34% to 42% (*p* < 0.0001), total motility rose from 37% to 45% (*p* <0.0001), concentration increased from 6.9 mil/mL to 9 mil/mL (*p* <0.0001), and morphology increased from 1% to 2% (*p* = .0026). Viability increased from 60% to 62% (p = .0002). At 6–12 months, number of motile sperm (7 mil/mL, *p* < 0.0001), progressive motility (38.5%, *p* = 0.0005), sperm concentration (8 mil/mL, *p* < 0.0001), and total motility (41.5%, *p* < 0.0001) remained statistically significant compared to baseline. At 12–18 months, significant increases in progressive motility (35%, *p* = 0.0272) and total motility (37%, *p* = 0.0022) persisted.

**Conclusions:**

Our retrospective study demonstrates significant short‐term improvements across multiple parameters at 6‐months after varicocoelectomy. While we note individual improvement in sperm parameters during longer follow‐up period, there was variability based on the time frame. Our findings underscore the impact of varicocoelectomies may have on enhancing short‐term male fertility. Future, prospective research is needed to validate these findings.

## 1 INTRODUCTION

Varicocoeles are found in 15%–20% of men, making it one of the most common male urologic conditions.^1^ While many patients may present asymptomatically, larger varicocoeles can cause dull pain or scrotal discomfort.^1^, ^2^ Varicocoeles have also been linked to male infertility with approximately 40% of these men being diagnosed with a varicocoele. Varicocoeles may contribute to infertility as a result of increased heat retention within the scrotum from pooling of blood, increasing oxidative stress and damaging sperm DNA.^3^, ^4^ Previous work has found decreased sperm motility, low sperm count, and abnormal sperm morphology in infertile men with varicocoeles.^5^ Therefore, varicocoelectomy—a procedure that re‐establishes normal flow in the pampiniform plexus—has emerged as the gold standard for treating varicocoele‐related infertility.^2^ 

Previous work has shown that varicocoelectomy improves sperm motility, count and morphology, as well as increase the chances of achieving pregnancy.^6^, ^7^ Gokce et al. reported that performing varicocoelectomy prior to intracytoplasmic sperm injection (ICSI) in 306 non‐azoospermic men significantly improved average total sperm count (16.2–35.4 mil/mL), total motile count (5.5–19.4 mil/mL), and sperm concentration (5.3–16.1 mil/mL) measured at three months post‐operatively, compared with men who did not undergo varicocoelectomy. This study also found a statistically significant increase in viable pregnancy and live births compared to controls.^6^

In a meta‐analysis of 35 randomized control trials by Wang et al. comparing the inguinal and subinguinal microsurgical techniques for varicocoelectomy, both strategies significantly improved sperm motility and concentration. The inguinal microsurgery method led to an increase in sperm density by 10.6 mil/mL and sperm motility by 9.09%. Again, varicocoelectomy was associated with significantly higher odds of achieving pregnancy compared to expectant therapy. Another meta‐analysis by Schauer et al. included 14 studies with a total of 1157 patients, demonstrating that varicocoelectomy significantly improves sperm count and motility, regardless of surgical technique.  However, long‐term studies evaluating whether these improvements persist over time remain limited. To address this gap, we retrospectively analyzed pre‐operative semen parameters and compared them with multiple post‐varicocoelectomy semen analyses (SA). Our objective was to assess trends across SA to determine whether parameters of male fertility continue to improve in the months following varicocoelectomy.

## 2 METHODS

 This study was approved by our Institutional Review Board #20170849. We conducted a retrospective chart review of male patients over the age of 18 who underwent microscopic subinguinal varicocoelectomy performed by a single surgeon between 1st January, 2017 and 31st December, 2023. Patients were identified using varicocoelectomy Current Procedural Terminology (CPT) codes 55520, 55530, 55535, or 55540 and manually chart reviewed for accuracy. Exclusion criteria consisted of patients who were under 18 years old or incarcerated. All men included had at least one pre‐operative and post‐operative SA. We collected data such as the number of motile sperm, progressive motility, round cells, semen volume, sperm concentration and morphology, total motility, and viability from each patient's SA. If a patient's semen analysis was missing data, those variables were coded as “NA” and excluded from analysis.

The most recent SA obtained prior to surgery for all patients was selected to serve as the patient's baseline parameters. Follow up SA were taken around 6, 12, 18, and 24 months after varicocoelectomy. For analysis, we performed the Shapiro–Wilk test and Wilcoxon tests. The Shapiro–Wilk test, along with the respective Q‐Q plots and histograms, was used to assess the normality of the paired differences in each variable. We used Wilcoxon signed‐rank tests to compare the difference in median values of each variable at baseline to respective parameters at each subsequent follow‐up time available after varicocoelectomy (Table 1). Additionally, we used Wilcoxon rank sum tests to compare differences in initial SA based on varicocoele laterality. Patients with a missing SA at a subsequent time period were excluded from that specific sub‐analysis. We performed a retrospective sample size analysis using the G*Power software. For a statistical power of 80%, the minimum sample size required would be 181 participants to detect a small significant effect. The Statistical Analysis System version 9.4 (SAS Institute) was used for statistical analysis, with significance set to *p *< 0.05.

**TABLE 1:** Semen analysis parameters before (“initial”) and after varicocoelectomy.

Parameter	Initial	0–6 months	6–12 months	12–18 months	18–24 months
**Number of Motile Sperm (millions)**					
**Median**	4	8	7	5.5	3.5
**IQR**	(1, 11)	(2, 18)	(0.9, 17)	(0.5, 14)	(0.1, 15.5)
**N**	252	295	127	43	23
***p*‐value**	–	**<0.0001**	**<0.0001**	0.1535	0.2661
**Progressive Motility (%) **					
**Median**	34	42	38.5	33	32
**IQR**	(19, 50)	(28, 54)	(23.5, 53.5)	(10, 49)	(12, 43)
**N**	234	279	115	38	21
***p*‐value**	–	**<0.0001**	**0.0005**	**0.0272**	0.457
**Round Cells (mil/mL)** ^b^					
**Median**	0.1	0.1	0.1	0.1	0.1
**IQR**	–	–	–	–	–
**N**	247	297	128	43	24
***p*‐value**	–	0.4409	0.3492	0.8203	0.625
**Semen Volume (mL)**					
**Median**	2.4	2.5	2.7	2.1	2.7
**IQR**	(1.6, 3.5)	(1.6, 3.5)	(1.7, 3.5)	(1.5, 3.0)	(2.1, 3.2)
**N**	263	286	123	42	22
***p*‐value**	–	0.1955	0.0876	0.9833	0.6407
**Sperm Concentration (mil/mL)**					
**Median**	6.9	9	8	8	3
**IQR**	(2, 13)	(3, 17)	(2, 16)	(2, 20)	(0.2, 13)
**N**	261	294	130	43	22
***p*‐value**	–	**<0.0001**	**<0.0001**	0.1108	0.2729
**Sperm Morphology** **(%)**					
**Median**	1	2	1	1	1.5
**IQR**	(0, 2.8)	(1, 4)	(0, 2)	(0, 2.5)	(0, 2)
**N**	185	184	24	14	6
***p*‐value**	–	**0.0026**	0.9902	0.75	1
**Total Motility (PR+NP, %)**					
**Median**	37	45	41.5	37	33
**IQR**	(22, 51)	(32, 54)	(30.5, 56)	(24, 51)	(15, 46)
**N**	241	279	115	38	22
***p*‐value**	–	**<0.0001**	**<0.0001**	**0.0022**	0.436
**Viability (live sperm, %)**					
**Median**	60	62	60.5	41.5	49.5
**IQR**	(47, 71)	(53, 71)	(45, 73.5)	(16.5, 76)	(18, 53)
**N**	188	184	27	14	6
***p*‐value**	–	**0.0002**	0.6775	0.625	0.125

^a^ Each subsequent data point was compared to initial value.

^b^ Round cells < 1 was considered 0.1 for the purposes of analysis.

## 3 RESULTS

We identified 328 patients (mean age 34.6 years) who underwent varicocoelectomy for this study using CPT code; participant characteristics may be found in Tables 2 and 3. Time of baseline semen analyses results prior to varicocoelectomy ranged from the same day to 40 months, with a mean of three and a half months. There were no differences in baseline semen parameters based on laterality (*p* > 0.05 for all parameters). Amongst the patients who followed up within 6 months after varicocoelectomy, we found improvement in number of motile sperm from 4 million (*n* = 252) to 8 million (*n* = 295, *p* < 0.0001), sperm concentration from 6.9 million/mL (*n *= 261) to 9 million/mL (*n* = 294, *p* < .0001), progressive motility from 34% (*n* = 234) to 42% (*n* = 279, *p* < 0.0001), and total motility from 37% (*n* = 241) to 45% (*n* = 279, *p* < 0.0001). Additionally, sperm morphology improved from 1% (*n* = 185) to 2% (*n* = 184, *p* = 0.0026) and viability increased from 60% (*n* = 188) to 62% (*n* = 184, *p* = 0.0002).

**TABLE 2:** Participant varicocoele laterality and grade.

Characteristic	Total *N* = 328
*Unilateral*	*n* = 161
L total	160
L 0	4
L I	22
L II	76
L III	58
R total	1
R 0	1
R I	0
R II	0
R III	0
*Bilateral*	*n* = 167
L total	167
L 0	6
L I	15
L II	77
L III	69
R total	167
R 0	6
R I	47
R II	96
R III	18

Abbreviations: L, left.; R, right.

**TABLE 3:** Descriptive statistics of participant baseline characteristics.

Value	Age	Left Grade	Right Grade
*Mean*	34.6	2.2	1.7
*Median*	34.0	2.0	2.0
*Mode*	31.0	2.0	2.0

In the 6–12 month post‐operative sub‐analysis (Table 1), number of motile sperm (7 mil/mL, *n* = 127, *p *< 0.0001), progressive motility (38.5%, *n *= 115, *p *= 0.0005), sperm concentration (8 mil/mL, *n* = 130, *p* < 0.0001), and total motility (41.5%, *n* = 115, *p* < 0.0001) remained significantly higher than the respective values at baseline. Additionally, between 12 and 18 months after varicocoelectomy, progressive motility (35%, *p* = .0272) and total motility (37%, *p* = .0022) remained elevated in comparison to initial SA. Trends in sperm concentration and number of motile sperm persisted, but these were not significantly different than baseline (*p *= 0.111, 0.153, respectively). Sperm morphology and viability were not statistically significant from baseline during this timeframe (*p *= 0.750, 0.625, respectively). There were no significant changes at any time point for semen volume and round cells. Finally, patients who followed up between 18 and 24 months saw no significant changes in any parameters compared to baseline.

## 4 DISCUSSION

Varicocoeles are a common cause of male infertility, mainly due to their negative impact on semen parameters and testicular function.^8^ However, despite this impact, there is limited information on the long‐term effects of varicocoelectomy on semen parameters. Our study adds to this body of literature by investigating changes in semen parameters after varicocoelectomy for up to 2 years after the procedure. Understanding these trends is important because it helps physicians better counsel patients on expectations after surgery.

In this study, we reviewed data from men who had a varicocoelectomy and compared their SA before surgery to multiple time points following surgery. We discovered that the most significant improvement in semen quality occurred within the first 6 months post varicocoelectomy. Such improvements included average number of motile sperm, sperm concentration, and progressive motility. These early changes suggest that varicocoelectomy quickly reduces the harmful effects of varicocoeles, such as increased scrotal temperature and oxidative stress, which can impair sperm quality.^9^ With the exception of viability and morphology, parameters that differed significantly from baseline at 0–6 months remained significantly different between 6 and 12 months.

After 12 months, the changes in semen quality were less consistent. Between 12 and 18 months, progressive motility and total motility continued to rise, but other parameters, including sperm morphology and viability, were unchanged from baseline levels. By 18–24 months, we did not find significant differences in any variable compared to initial SA. However, only 43 and 24 men, respectively, completed SA at these time points, and our analysis is limited; it is possible that these men may have only followed up due to ongoing issues with conceiving. Overall, while some improvements continued beyond six months, the data show that the fertility benefits of varicocoelectomy may be marginal after this initial period.

These results align with previous literature that show early improvements in semen quality post varicocoelectomy. In a retrospective review of 100 men after varicocoelectomy for subfertility, Bakri et al. demonstrated that the majority of improvements occur within the first 3 months, with minimal changes thereafter.^10^ A meta‐analysis of four randomized controlled trials and 44 prospective studies performed by Baazeem et al. reported significant increases in sperm concentration and motility at 3–6 month following varicocoelectomy.^11^ As mentioned, all varicocoelectomies in our study were completed via the subinguinal approach, which has been shown to equally improve sperm parameters compared to other techniques. Specifically, work by Schauer in 2012 demonstrated that high ligation surgery increased sperm count by 10.85 million/mL and motility by 6.80%, inguinal varicocoelectomy improved sperm count by 7.17 million/ml and motility by 9.44%, and subinguinal varicocoelectomy increased sperm count by 9.75 million/mL and motility by 12.25%. The inguinal approach was associated with the highest pregnancy rate of 41.48%, compared to 26.90% and 26.56% for high ligation and subinguinal varicocoelectomy, respectively. Of note, this meta‐analysis specifically uses the term sperm count, which equates to sperm concentration as defined at our institution.^12^

Previous work has shown that improved semen parameter outcomes after varicocoelectomy may not persist in the long term, which is corroborated by our findings. A 2024 meta‐analysis (*N* = 4) by Mei et al states that semen analyses were optimal quite early after varicocoelectomy. While this is consistent with our results, this study analyzed SA results on even smaller time scale than we did, discovering that SA parameters were best approximately 3 months after varicocoelectomy, even when compared to six months or more.^13^ Another study by Pallotti et al. noted that while motility and concentration improved significantly after surgery, some parameters, such as morphology, demonstrated inconsistent trends over time.^14^ This variability reflects the complex interaction between factors such as semen quality, varicocoele grade, and post‐surgical recovery. Furthermore, Kızılay et al. highlighted the potential influence of adjunctive treatments, such as lifestyle modifications or antioxidant therapy, in sustaining long‐term improvements in semen quality after varicocoelectomy.^15^

There are some limitations to our study. Given the retrospective nature of our work, we could not control for all factors that might affect semen quality, like smoking or past medical conditions.^14^ Also, very few patients followed up at longer time points. For example, between 6 and 24 men followed up after 18 months, which makes it more difficult to have adequately powered sub analyses and draw conclusions about the long‐term effects of the surgery.^10^ Future studies could examine serial semen analyses on a monthly (e.g. 0–3 months, 3–6 months) or perhaps weekly basis to gather a robust database and delineate specific time ranges where parameters begin to decline. These studies must also include larger groups of patients and follow them prospectively over time to support our findings.^16^  It would also be helpful to investigate other treatments during the post‐operative setting, like antioxidant supplements, that might support long‐term improvements after varicocoelectomy.^15^ Finally, studying pregnancy outcomes in the setting of varicocoelectomy would give us a better understanding of how this surgery impacts fertility.^11^ Overall, given our large single‐surgeon study with longitudinal data, our findings contribute to the growing body of evidence supporting varicocoelectomy as an effective intervention for male infertility.

## 5 CONCLUSIONS

Our retrospective study highlights the significant effects of varicocoelectomy on semen analysis parameters in men with varicocoeles, which affect a large proportion of the adult male with infertility. Our findings demonstrate that varicocoelectomy provides meaningful short‐term improvements in semen quality—most notably in number of motile sperm, sperm concentration, progressive motility and total motility. Furthermore, while some parameters showed variability at later time points, there were still significant improvements in semen analysis parameters, such as number of motile sperm at 12–18 months and round cells at 18–24 months. These findings highlight the efficacy of varicocoelectomy in potentially mitigating varicocoele‐related infertility. Future prospective studies should utilize larger cohorts, standardized follow‐up protocols, and adjunctive therapies to optimize and prolong these observed benefits. Ultimately, our results underscore the importance of varicocoelectomy in managing male infertility, while emphasizing the need for ongoing research to elucidate the long‐term impact on reproductive outcomes.

## AUTHOR CONTRIBUTIONS

All authors meets the ICMJE criteria for authorship. **Max D. Sandler**: Methodology; validation; formal analysis; investigation; data curation; writing—original draft; writing—review & editing; visualization; supervision; project administration. **Juli o Yanes**: Methodology; validation; formal analysis; investigation; data curation; writing—original draft; writing—review & editing. **Rohan Dureja**: Methodology; validation; formal analysis; investigation; data curation; writing—original draft; writing—review & editing. **Vishal Ila**: Conceptualization; methodology; validation; formal analysis; investigation; data curation; writing—review & editing; supervision. **Aaron Gurayah**: Conceptualization; methodology; validation; formal analysis; investigation; data curation; writing—original draft; writing—review & editing; visualization; supervision; project administration. **Adam D. Williams**: Investigation; formal analysis; data curation; writing—review & editing; visualization. **David Miller**: Conceptualization; methodology; validation; formal analysis; investigation; data curation; writing—original draft; writing‐ review & editing; visualization; supervision; project administration.

## CONFLICT OF INTEREST STATEMENT

The authors declare no conflicts of interest.

## Data Availability

The data that support the findings of this study are available on request from the corresponding author. The data are not publicly available due to privacy or ethical restrictions.
